# Adenosine Receptors Expression in Human Retina and Choroid with Age-related Macular Degeneration

**DOI:** 10.18502/jovr.v18i1.12725

**Published:** 2023-02-21

**Authors:** Collin P. Goebel, Yong-Seok Song, Ismail S. Zaitoun, Shoujian Wang, Heather A. D. Potter, Christine M. Sorenson, Nader Sheibani

**Affiliations:** ^1^Department of Ophthalmology and Visual Sciences, University of Wisconsin School of Medicine and Public Health, Madison, WI, USA; ^2^McPherson Eye Research Institute, University of Wisconsin School of Medicine and Public Health, Madison, WI, USA; ^3^Department of Cell and Regenerative Biology, University of Wisconsin School of Medicine and Public Health, Madison, WI, USA; ^4^Department of Biomedical Engineering, University of Wisconsin, Madison, WI, USA

**Keywords:** Caffeine, Choroid, Inflammation, Neovascularization, Neurodegeneration, Retina

## Abstract

**Purpose:**

Adenosine signaling modulates ocular inflammatory processes, and its antagonism mitigates neovascularization in both newborns and preclinical models of ocular neovascularization including age-related macular degeneration (AMD). The adenosine receptor expression patterns have not been well characterized in the human retina and choroid.

**Methods:**

Here we examined the expression of adenosine receptor subtypes within the retina and choroid of human donor eyes with and without AMD. Antibodies specifically targeting adenosine receptor subtypes A1, A2A, A2B, and A3 were used to assess their expression patterns. Quantitative real-time PCR analysis was used to confirm gene expression of these receptors within the normal human retina and choroid.

**Results:**

We found that all four receptor subtypes were expressed in several layers of the retina, and within the retinal pigment epithelium and choroid. The expression of A1 receptors was more prominent in the inner and outer plexiform layers, where microglia normally reside, and supported by RNA expression in the retina. A2A and A2B showed similar expression patterns with prominent expression in the vasculature and retinal pigment epithelium. No dramatic differences in expression of these receptors were observed in eyes from patients with dry or wet AMD compared to control, with the exception A3 receptors. Eyes with dry AMD lost expression of A3 in the photoreceptor outer segments compared with eyes from control or wet AMD.

**Conclusion:**

The ocular presence of adenosine receptors is consistent with their proposed role in modulation of inflammation in both the retina and choroid, and their potential targeting for AMD treatment.

##  INTRODUCTION

Age-related macular degeneration (AMD) is an inflammatory driven neurodegenerative disorder that develops in the elderly due to a combination of genetics, the environment, and other factors. It contributes to substantial irreversible central vision loss in the industrialized countries and was present in an estimated 6.5% of all American adults over the age of 40 as of 2011.^[1]^ AMD is characterized by the deposition of cellular debris, called drusen between the retina and choroid, and divided into the categories of wet and dry AMD.^[2]^ In patients with wet AMD, subretinal neovascularization occurs, which may lead to edema and hemorrhage. In contrast, dry AMD is characterized by drusen and retinal degeneration without neovascularization or hemorrhage. Currently, treatment for AMD, especially dry AMD, is very limited. Antibodies against vascular endothelial growth factor (VEGF) improve visual outcomes in most patients with wet AMD.^[3,4]^ In addition, antioxidant vitamins and minerals slow down the progression of moderate or severe dry AMD but fail to prevent the development of moderate AMD from mild AMD.^[5]^ Thus, these therapeutics are best for preventing progression in patients who have already developed moderate or severe disease and are unable to halt or reverse the disease process.

To develop better therapeutic targets for patients with AMD, a more complete understanding of its pathophysiology is required. The dysfunction and death of retinal pigment epithelial (RPE) cells and degeneration of photoreceptors are observed in AMD. However, the detailed cellular and molecular mechanisms driving this degeneration needs further investigation. Several hypotheses have been proposed, including accumulation of toxins, dysfunction of mitochondria, and damage from reactive oxygen species in the RPE cells and choroidal vasculature.^[5,6,7,8]^ Recently, immune and inflammatory regulatory pathways have been proposed to be the central components in the pathophysiology of AMD, and preclinical models have demonstrated complement and IgG deposition in the RPE and choroid of mice with AMD.^[5,8,9,10]^

Recent investigations indicate the importance of adenosine receptors in modulation of ocular inflammatory processes. Adenosine elicits its effects through its G-protein coupled receptors: A1, A2A, A2B, and A3.^[11]^ Adenosine receptor A1 activation inhibits calcium influx-induced release of neurotransmitters in the central nervous system (CNS) under hypoxic conditions, creating a neuroprotective effect.^[12]^ Adenosine receptor A3 engagement has both pro- and anti-inflammatory properties throughout the body, including the lung, cardiac, and gastrointestinal systems.^[10]^ Blockade of the adenosine A2A receptor in microglial cells reduces inflammatory responses and photoreceptor cell loss in cultured human cells. Furthermore, adenosine receptors A2A and A2B expression are upregulated by hypoxia-inducible factor during hypoxic conditions and inflammation in the eye,^[13,14]^ and their antagonism blocks ischemia-mediated retinal neovascularization.^[15]^ Thus, the process of inflammation and angiogenesis in dry and wet AMD could be linked with adenosine receptor signaling. However, the function of these receptors in the RPE and choroid, and their potential activity in pathophysiology of AMD, needs further evaluation.

The importance of adenosine receptor signaling pathways in ocular inflammatory and neovascular diseases has been further supported by studies of caffeine, an adenosine receptor antagonist, in both preclinical models and humans.^[16]^ Maugeri and colleagues found evidence that caffeine decreases the permeability of the RPE layer and thus may inhibit the development of macular edema.^[17]^ In addition, caffeine administered to infants born prematurely for apnea diminishes the severity of retinopathy of prematurity.^[18]^ We recently showed that caffeine is efficacious in mitigating choroidal neovascularization in a preclinical model of wet AMD.^[19]^ However, the identity of adenosine receptor(s) involved in these activities remains unknown, and results have yet to be verified in humans. Here we assessed the presence of specific adenosine receptors in the retina and choroid of human donor eye samples from control and patients with wet and dry AMD. These studies, to the best of our knowledge, are the first to demonstrate the presence of specific adenosine receptors in the human retina and choroid and examine whether their expression pattern is altered under AMD conditions.

##  METHODS

### Human Donor Eyes and Other Materials

Deidentified human ocular samples were from the Lion Gift of Sight (St. Paul, MN). The eyes were collected by written consent from donors or donors' family for medical research as delineated by the Declaration of Helsinki. We were provided with a list of 28 potential donor samples with histological evaluations, of which 13 were control eyes and 15 were eyes with AMD. Eyes from two donors with wet AMD, two donors with dry AMD, and two donors with no AMD were selected by the help of our ocular pathologist from the available samples. Each experimental group contained samples matched by age and gender, and all samples included the macula. Presumptive diagnoses were confirmed histologically. Anti-ADORA1 (55026-1-AP, Proteintech, Rosemont, IL), anti-ADORA2A (PA1-042), anti-ADORA2B (PA5-72850), and anti-ADORA3 (PA5-36350) were obtained from Thermo Fisher Scientific (Carlsbad, CA). Anti-collagen IV antibody was from Southern Biotech (1340-01; Birmingham, AL). Cy5-labeled anti-goat (705-175-147) and Cy2-labelled anti-rabbit (305-225-045) were obtained from Jackson ImmunoResearch Laboratories (West Grove, PA).

### Antibody Staining and Microscopic Analysis of Eye Sections

Four paraffin sections, taken from each donor eye, were placed on glass slides. Sections were washed with xylene four times for five min. This was followed by two washes in 100% and 95% ethanol for 10 min, and the pure water for 5 min. Slides were then heated in a citrate solution (H-3300, Vector Laboratories, Burlingame, CA) for 11 min to retrieve epitopes. For each set of samples, slides were then stained overnight with 750 μ L ADORA1, ADORA2A, ADORA2B, and ADORA3 primary antibodies, diluted in blocking buffer (1:500; PBS with 1% bovine serum albumin, 0.2% skim milk powder, and 0.3% Triton-X100). Diluted Anti-collagen IV antibody (1:500) was added to each sample to target vasculature in the samples, and DAPI diluted 1:1000 was added to each sample to visualize the cellular nuclei of the retina and choroid. The slides were then rinsed with PBS buffer three times for 5 min, and 750 μ L of appropriate secondary antibodies (diluted 1:500 in PBS blocking buffer) were added to each sample. Slides were incubated at room temperature for 4 h allowing the visualization of collagen in the vasculature and adenosine receptor expression.

Following staining with primary and secondary antibodies, the expression of the A1, 2A, 2B, and A3 receptors in donor eyes with wet AMD, dry AMD, or no AMD were compared using fluorescence microscopy. Light intensity and exposure time were standardized for each group of slides under the microscope. Photographs were taken of fluorescence emission patterns for the adenosine receptor, collagen IV, and DAPI located within the macula and underlying choroid. The fluorescence intensity in each sample was then compared to determine the predominant location of each adenosine receptor in the retina and choroid, as well as look for differences in adenosine receptor expression between wet and dry AMD compared to control.

### RNA Isolation and Quantitative PCR (qPCR) Analysis

The retina and RPE/choroid were dissected from at least three non-diseased human eyes of similar age (male and female) and cut into smaller pieces in cold PBS. The tissue samples were snap frozen in liquid nitrogen and stored at –80ºC for RNA preparation. Tissue samples (50–100 mg) were dissolved in 1 mL of Trizol reagent (Invitrogen, San Diego, CA). Total RNA was extracted using RNeasy mini kit as recommended (Qiagen, Valencia, CA). Complementary DNA was prepared using 1 μg of total RNA and the RNA to cDNA EcoDry Premix (TaKaRa, Mountain View, CA) and diluted 1:10. qPCR was conducted in triplicates using a Mastercycler Realplex (Eppendorf, Enfield, CT) and TB-Green qPCR Premix (TaKaRa). The cycles for amplification were 95ºC for 2 min; 40 cycles of amplification (95ºC for 15 s, 60ºC for 40 s); and dissociation curve step (95ºC for 15 s, 60ºC for 15 s, 95ºC for 15 s). The relative fluorescent units (RFUs) at a threshold fluorescence value (Ct) were used for linear regression line and assessment of nanograms of DNA. The target gene expression levels were determined by comparing the RFU at the Ct to the standard curve and normalized by the housekeeping gene ribosomal protein L13α (RPL13A). The primer sequences used in this study are listed in Table 1. Each sample was run in triplicates.

### Statistical Analysis

Differences between the expression level of ADORA in the retina and RPE/Choroid were evaluated using *t*-tests and GraphPad Prism version 8 (GraphPad Software, La Jolla, CA). *P* < 0.05 was considered significant. Data are the mean ± standard deviation.

**Figure 1:**
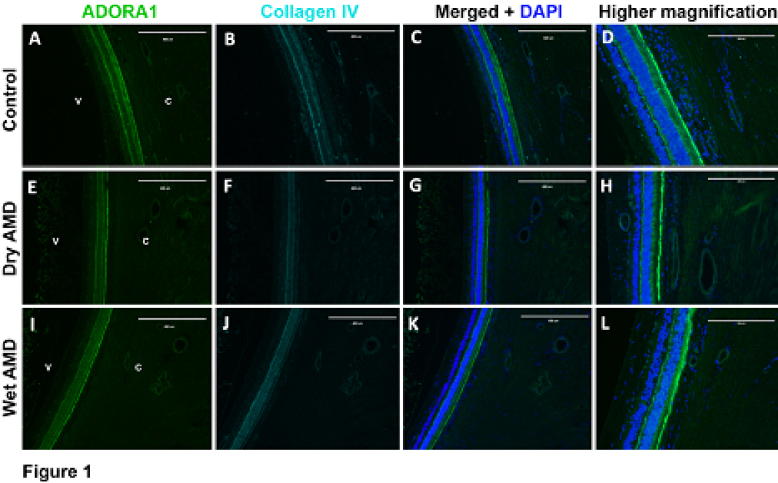
Expression ofADORA1 in retinal and choroidal cross sections. (A) ADORA1, (B) collagen IV, and (C) merged images and DAPI staining of eye sections with no AMD (Control). (D) A higher magnification of C. (E) ADORA1, (F) collagen IV, and (G) merged images and DAPI staining of eye sections with dry AMD. (H) A higher magnification of G. (I) ADORA1, (J) collagen IV, and (K) merged images and DAPI staining of eye sections with wet AMD. (L) Higher magnification of K. Scale bar = 400 µm (A, B, C, E, F, G, I, J, and K) and 200 µm (D, H, and L). V, vitreous; C, choroid.

**Figure 2:**
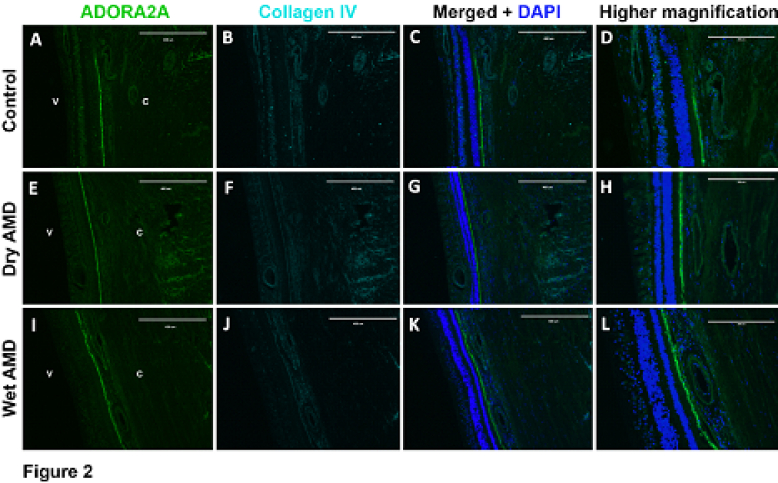
Expression ofADORA2A in retinal and choroidal cross sections. (A) ADORA2A, (B) collagen IV, (C) merged images and DAPI staining of eye sections with no AMD (Control). (D) A higher magnification of C. (E) ADORA2A, (F) collagen IV, and (G) merged images and DAPI staining of eye sections with dry AMD. (H) A higher magnification of G. (I) ADORA2A, (J) collagen IV, and (K) merged images and DAPI staining of eye sections with wet AMD. (L) A higher magnification of K. Scale bar = 400 µm (A, B, C, E, F, G, I, J, and K) and 200 µm (D, H, and L). V, vitreous; C, choroid.

**Figure 3:**
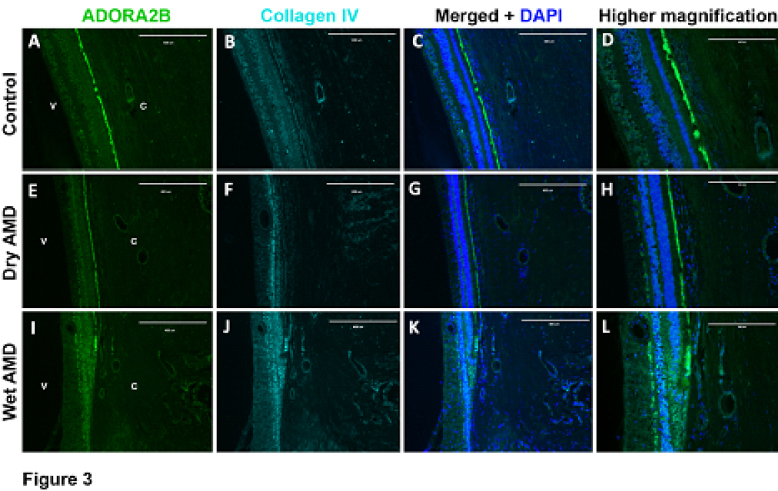
Expression ofADORA2B in retinal and choroidal cross sections. (A) ADORA2B, (B) collagen IV, and (C) merged images and DAPI staining of eye sections with no AMD (Control). (D) A higher magnification of C. (E) ADORA2B, (F) collagen IV, (G) merged images and DAPI staining of eye sections with dry AMD. (H) A higher magnification of G. (I) ADORA2B, (J) collagen IV, and (K) merged images and DAPI staining of eye sections with wet AMD. (L) A higher magnification of K. Scale bar = 400 µm (A, B, C, E, F, G, I, J, and K) and 200 µm (D, H, and L). V, vitreous; C, choroid.

**Figure 4:**
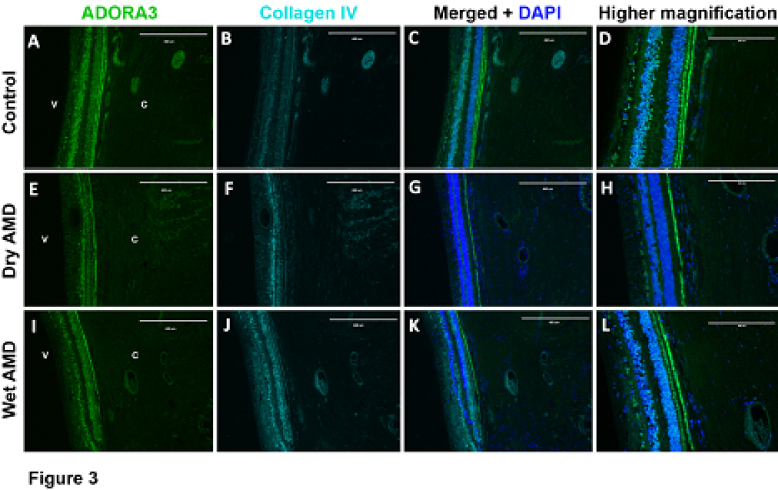
Expression ofADORA3 in retinal and choroidal cross sections. (A) ADORA3, (B) collagen IV, and (C) merged images and DAPI staining of eye sections with no AMD (Control). (D) Higher magnification of C. (E) ADORA3, (F) collagen IV, and (G) merged images and DAPI staining of eye sections with dry AMD. (H) higher magnification of F. (I) ADORA3, (J) collagen IV, and (K) merged images and DAPI staining of eye sections with wet AMD. (L) Higher magnification of K. Scale bar = 400 µm (A, B, C, E, F, G, I, J, and K) and 200 µm (D, H, and L). V, vitreous; C, choroid.

**Figure 5:**
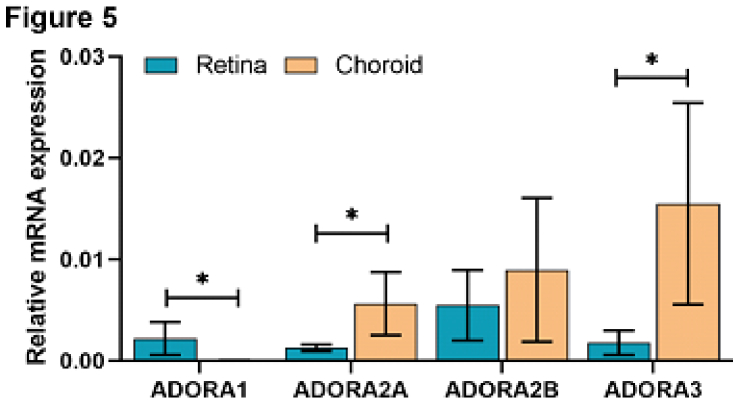
Quantitative PCR results demonstrating relative mRNA expression of adenosine receptor subtypes in a human eye sample without AMD. Blue bars display mRNA expression for each receptor in the retina, and orange bars display mRNA expression for each receptor in the choroid. ADORA1 and ADORA2B showed significantly higher levels in the retina, while ADORA2A and ADORA3 showed significantly higher levels in the choroid. *P < 0.05, *n* = 3.

**Table 1:** List of primers.


orange**Gene**	orange**Forward (5' to 3')**	orange**Reverse (5' to 3')**
ADORA1	gtccggtcctcatcctcac	ccaccatcttgtaccggaga
ADORA2A	cacaccactctccctagactctc	ttcctcacacttacatttttcctg
ADORA2B	cactgcttataatgctggtgatcta	gggtggtcctcgagtggt
ADORA3	cccaattatatctcccccact	aagtcaggcctccaaaacact
RPL13A	aagcggatgaacaccaacc	tgtggggcagcatacctc
	
	

##  RESULTS

### Adenosine Receptors Expression in Retina and Choroid

Each eye section selected for fluorescent staining was from a patient between the ages of 76 and 100 years old at the time of death. One male and one female sample was chosen for each of the AMD and control groups. Each sample was preserved within 28 h of the patient's death. All samples demonstrated successful staining with each of the adenosine receptor antibodies. Furthermore, vascular staining with collagen IV antibody and nuclear staining with DAPI were performed for each of the samples.

Adenosine receptor A1 demonstrated expression throughout the retina. A1 expression was particularly prominent in the outer plexiform layer (OPL), inner photoreceptor layer, and inner plexiform layer (IPL) of the retina. Retinal pigment epithelium (RPE) was also positive. No noticeable differences in choroidal vascular or retinal expression of the A1 were appreciated between patients with AMD compared to control patients regardless of sex. Representative images of A1 receptor staining in the retina and choroid are shown in Figure 1.

Receptor A2A primarily demonstrated expression within the retinal and choroidal vasculature, with a modest expression within the outer (ONL) and inner nuclear layers (INLs). The expression of the A2A was also detected in the RPE and was similar in patients with AMD compared to control eyes. However, there did appear to be a modest decrease in A2A receptor expression in retinal and choroidal vasculature in samples from patients with wet and dry AMD. There did not appear to be a dramatic difference in receptor A2A expression between patients with wet AMD compared with dry AMD [Figure 2].

Receptor A2B demonstrated expression throughout the retina in all samples, particularly the ganglion cell layer, ONL, and INL, and RPE. Receptor A2B was also strongly expressed in the retinal and choroidal vasculature in all samples. However, there was no dramatic difference in A2B receptor staining in the retina, retinal vasculature, or choroidal vasculature of patients with AMD compared with control eyes. Representative images of retinal and choroidal staining with antibodies to the A2B receptor are shown in Figure 3.

Receptor A3 was expressed primarily within the ganglion cell layer, INL, and ONL of the retina, and RPE. There was also some A3 receptor expression in the retinal and choroidal vasculature of each sample. A dramatic differences in A3 staining was observed in dry AMD samples compared with control and wet AMD samples. The dry AMD samples lost the expression of A3 receptor in the photoreceptor outer segments, which was prominently present in control and wet AMD samples. However, no additional differences in the intensity of staining were observed in retina or choroid in patients with AMD compared to those with no AMD. Representative images of A3 receptor staining are shown in Figure 4.

### Adenosine Receptors mRNA Expression in Retina and RPE/Choroid Tissues

Quantitative PCR of cDNA prepared from the retinal and choroidal/RPE tissues from normal human eyes demonstrated notable expression of adenosine receptors within both the retina and choroid. Adenosine receptor A1 displayed gene expression primarily within the retina, with little to no choroidal expression. This was consistent with predominant immunostaining of A1 receptor in IPL and OPL, where microglia are normally residing. We previously showed predominant expression of A1 receptor in mouse microglia and retinal vascular cells.^[19]^ Receptors A2A, A2B, and A3 displayed gene expression within both the retina and choroid. Although A1 receptor expression was significantly lower, the expression of A2A and A3 were significantly higher in the choroid/RPE compared with the retina, as we previously reported in human and mouse tissue samples.^[19]^ The average relative expression of each adenosine receptor in both the retina and choroid/RPE is shown in Figure 5.

##  DISCUSSION

The role of adenosine receptors in inflammatory pathways, as well as prior clinical and preclinical studies of their antagonism with caffeine suggest that these receptors may play important roles in the development of neurodegenerative diseases such as AMD. However, previous literature has not sufficiently examined the distribution of the adenosine receptor subtypes in the human retina and choroid. Furthermore, this is the first study to compare the expression patterns of adenosine receptors in the eyes of patients with and without AMD.

Collectively, our qPCR and antibody staining experiments demonstrated that adenosine receptors are widely expressed throughout the human eye and are present within both the human retina and choroid. Antibody staining suggested that receptors A1, A2A, A2B, and A3 are widely expressed in multiple layers of the human retina, providing further support for the importance of these receptors in the human ocular homeostasis and pathophysiology of AMD. A recent study involving zebrafish studied the expression of all four adenosine receptor subtypes, reporting A2A and A2B receptors in both the inner and outer plexiform and nuclear layers, and the ganglion cell layer.^[20]^ Our results demonstrated similar distribution of the A2B receptors throughout the retina, but we primarily observed the A2A receptors expressed within the vasculature. Much like our results, A3 receptors were primarily in the inner and ONLs and A1 receptors were primarily in the inner and OPLs.^[20]^ Therefore, our results indicate some overlap with the preclinical models mapping the expression of adenosine receptors in the retina, but we did find differences in human retinal expression compared to animal models.

Prior human studies have examined the location of adenosine A1 receptors in the retina of humans and other mammals. Like our results, these studies demonstrated A1 expression in both the inner and outer retina, with expression in the inner plexiform, ganglion cell, inner nuclear, and photoreceptor layers.^[21,22]^ A separate study demonstrated the presence of A2 receptors within the human RPE but did not attempt to map the A2 receptors throughout the retina.^[23]^

In addition to the human neuroretina, receptors A2A, A2B, and A3 were strongly expressed within the retinal and choroidal vasculature. Studies have suggested that the loss of the inner choroidal vascular layer is associated with the development of AMD and likely to occur due to inflammation within the choroid.^[24]^ Our observation of adenosine receptors within the choroidal vasculature and their involvement in inflammation suggests they may also have a role in hallmark AMD changes within the choroid.

We hypothesized that altered A2A expression in patients with wet and dry AMD could contribute to pathophysiology of AMD. Prior studies have suggested that A2A receptor stimulation is pro-inflammatory, and antagonism of the A2A receptor can prevent neovascularization in the retina.^[13,14][15]^ Interestingly, our results suggest there may be a modest decrease in the expression of the A2A receptor in patients with AMD disease process, which requires further verification in future studies. Therefore, a change in the A2A receptor levels in the human retina and choroid may disrupt normal signaling in the eye potentially contributing to pathogenesis of AMD.

A previous study in our lab examined the effect of caffeine, an adenosine receptors antagonist, and istradefylline, a specific A2A receptor antagonist, on choroidal neovascularization after laser-induced rupture of Bruch's membrane. These studies demonstrated that antagonism of adenosine receptors, particularly A2A, was successful in inhibiting choroidal neovascularization. We also demonstrated that caffeine inhibits the migration of retinal and choroidal endothelial cells.^[19]^ Thus, the results of the current study suggesting that the A2A receptor may have altered expression in human eyes with AMD fits well with prior findings. Together these results suggest a potential role for adenosine receptor antagonism in preventing changes associated with AMD.

In our previous study we noted variable and limited expression of A3 receptor in retina and choroid/RPE tissues from mouse eyes.^[19]^ However, here we noted significant expression of A3 receptor in human eye sections with predominant expression in the choroid/RPE. The immune staining of the eye sections from dry AMD patients lacked A3 staining in the photoreceptor outer segments, which was predominantly present in eye sections from control and wet AMD patients. Thus, downregulation of A3 receptor expression may specifically contribute to loss of photoreceptor cells in dry AMD and awaits future studies of its significance in pathophysiology of dry AMD.

Overall, this study suggests that adenosine receptors are present throughout the retina and choroidal vasculature and supports the potential role of adenosine as a key signaling molecule and inflammatory mediator in pathophysiology of AMD. Furthermore, there may be changes in the levels of adenosine receptor A2A and A3 expression in patients who have AMD. However, the number of samples evaluated here were limited and awaits further confirmation of these results using additional samples in future studies as more suitable samples become available. We propose it is possible that the adenosine receptors contribute to the development of both wet and dry AMD and are suitable candidates to be targeted in the ongoing search for AMD therapeutics in future studies.

##  Ethical Considerations

The human eyes were obtained from Lions Gift of Sight (formerly known as Minnesota Lions Eye Bank, Saint Paul, MN) with the written consent of the donor or the donor's family for use in medical research in accordance with the Declaration of Helsinki. Lions Gift of Sight is licensed by the Eye Bank Association of America (accreditation #0015204) and accredited by the FDA (FDA Established Identifier 3000718528). Donor tissue is considered pathological specimens and is therefore exempt from the process of Institutional Review Board approval.

##  Acknowledgements

The authors would like to thank the Lion Gift of Sight (St. Paul, MN) personnel for obtaining the eyes and preparing the tissues for immunostaining. They are also thankful to the donors and their families for their valuable contributions to the research.

##  Financial Support and Sponsorship

This work and/or the investigator(s) were supported by an unrestricted award from Research to Prevent Blindness to the Department of Ophthalmology and Visual Sciences, Retina Research Foundation, RRF/Daniel M. Albert chair, and by National Institutes of Health grants P30 EY016665, R01 EY026078, EY030076, EY032543, and HL158073. CPG was recipient of a VitreoRetinal Surgery Foundation research award, Edina, MN.

##  Conflicts of Interest

The authors declare no conflict of interest.
